# Successful Imatinib Treatment for Systemic Mastocytosis Associated With Myelodysplastic/Myeloproliferative Neoplasm: Report of a Case and Literature Review

**DOI:** 10.3389/fonc.2021.819097

**Published:** 2022-01-12

**Authors:** Enrico Barozzi, Cristina Bucelli, Federica Irene Grifoni, Umberto Gianelli, Alessandra Iurlo, Daniele Cattaneo

**Affiliations:** ^1^Hematology Division, Foundation IRCCS Ca’ Granda Ospedale Maggiore Policlinico, Milan, Italy; ^2^Department of Oncology and Hemato-Oncology, University of Milan, Milan, Italy; ^3^Department of Pathophysiology and Transplantation, University of Milan, Milan, Italy; ^4^Division of Pathology, Foundation IRCCS Ca’ Granda Ospedale Maggiore Policlinico, Milan, Italy

**Keywords:** imatinib, systemic mastocytosis, SM-AHN, MDS/MPN, myeloid neoplasms

## Abstract

Systemic mastocytosis (SM) is a heterogeneous disease characterized by the expansion of mast cells in one or more tissues, frequently characterized by the presence of *KIT*D816V mutation. The updated World Health Organization (WHO) classification of myeloid neoplasms recognizes SM with an associated hematological neoplasm (SM-AHN) as a new subtype among the others, which is depicted by the coexistence of SM with another hematological clonal disease. Prognosis is very different among SM patients, while its treatment, although highly personalized, is still challenging. Here we report a case of *KIT*D816V-unmutated SM associated with MDS/MPN successfully treated with imatinib.

## Introduction

Systemic mastocytosis (SM) is a rare hematological neoplasm characterized by the abnormal proliferation and accumulation of mast cells (MCs). Clinical manifestations are heterogeneous depending on the tissue infiltration and MC mediators released by their degranulation (1). Minor diagnostic criteria include: (I) >25% of all MCs are atypical cells on bone marrow (BM) smears; (II) gain-of-function point mutations at codon 816 of *KIT* gene; (III) high serum tryptase level (unless there is an associated clonal myeloid disorder, in which case this parameter is not valid); and (IV) abnormal MCs CD25/CD2 expression. However, the unique major criterion is depicted by multifocal, dense infiltrates of MCs (≥15 MCs in aggregates) detected in sections of BM and/or other extracutaneous organs (2, 3). Accordingly, the diagnosis of SM can be made when the major criterion and one minor criterion or at least three minor criteria are present. In the WHO classification, SM is divided into: indolent SM; smoldering SM (SSM); SM with an associated hematological neoplasm (SM-AHN); aggressive SM; and MC leukemia (2, 3). Signs of an excessive MC burden in the tissue, called B-findings, and signs of specific organ damage, called C-findings, are used to define the different subgroups of SM (4). Regarding SM-AHN, whose diagnosis requires the presence of both SM criteria and WHO criteria for a clonal hematological neoplasm, SM is most frequently associated with myeloid malignancies such as myeloproliferative neoplasms (MPN), myelodysplasia (MDS), and MDS/MPN overlapping syndromes, such as chronic myelomonocytic leukemia (CMML). The goal of SM therapy is stratified according to the specific subtype. Indolent SM therapy is focused on symptom relief such as pruritus, flushing, gastrointestinal cramping, and osteoporosis, while in the aggressive forms the primary therapeutic target is the improvement of organ damage by means of cytoreductive and/or targeted therapies, including tyrosine-kinase inhibitors (1, 4–6).

## Case Description

**Figure 1:**
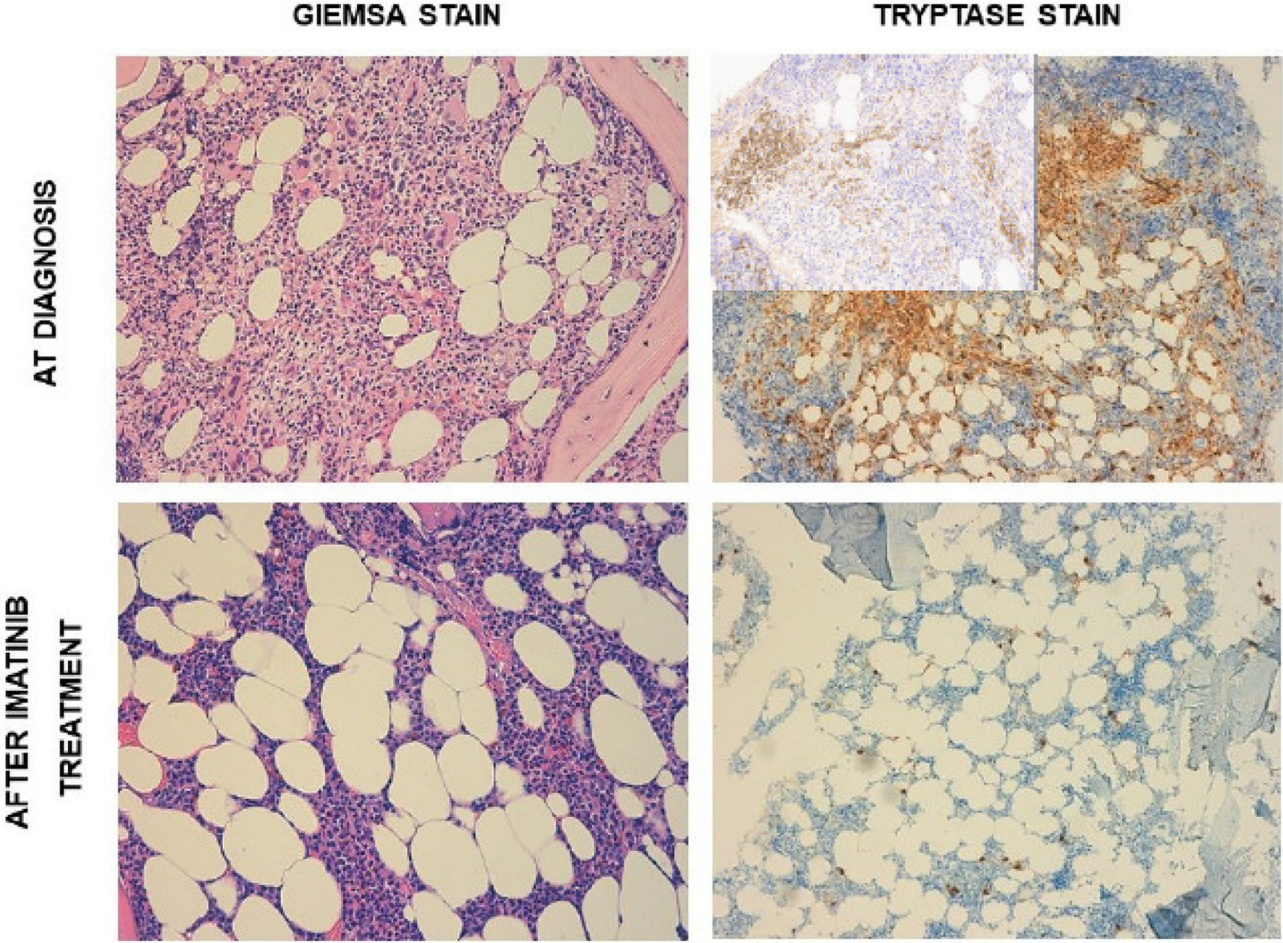
Upper quadrants: bone marrow morphology at diagnosis. Lower quadrants: bone marrow morphology after imatinib treatment (3 months). Left quadrants: Giemsa stain. Right quadrants: tryptase stain with CD117 immunohistochemistry (insert).

A 56-year-old Caucasian woman presented to our hospital with maculopapular rash of the chest, hepato-splenomegaly, and enlarged inguinal, axillary, and lateral cervical lymph nodes with a short axis less than 1 cm. Importantly, the patient suffered from different antibiotic allergies (namely, penicillin and vancomycin). Past medical history was remarkable for breast carcinoma treated in 2007 with radiotherapy and hormone therapy. In addition, two years before the first access to our hospital, the patient suffered from vertebral fracture initially interpreted as post-traumatic. Routine blood analysis revealed mild anemia (Hb 10.5 g/dl), white blood cells count (WBC) of 12.3 × 10^9^/L with significantly increased basophils (3.08 × 10^9^/L), and thrombocytosis (platelets count, 567 × 10^9^/L). Serum lactate dehydrogenase (LDH) level was 224 IU/L. Renal function tests were normal, and also coagulation, C-reactive protein, and liver function tests (including alkaline phosphates). Neither folic acid nor cobalamin deficiency was detected. The abdomen ultrasonography showed an enlarged spleen (diameter of 19 cm), while enlarged lymph nodes were defined as reactive. Screening for *JAK2*V617F, *CALR*, and *MPL* mutations, and *BCR-ABL1* p210, p190, and p230 fusion transcripts were all negative. Therefore, a BM aspirate and biopsy were performed, the first with characteristics of dyserithropoiesis with megaloblastic changes and of dismegakaryopoiesis with hypolobated and multinucleated megakaryocytes. BM examination revealed a myeloid/erythroid ratio of >5/1, and grade 2 reticulin fibrosis, loose and dense clusters of hyperlobulated or hypolobulated and multinucleated megakaryocytes, along with an infiltrate of approximately 30% of atypical MCs, which were tryptase+, CD117+/−, CD25+/−, and CD30−. Cytogenetic analysis revealed a normal female karyotype. Next-generation-sequencing test for myeloid genes (Illumina MiSeqTM) detected no mutations in any of the 30 genes analyzed, including *KIT*. Second-level analyses showed increased serum tryptase level (148 ng/ml) and peripheral CD34+ cells (172/µl). Thus, a diagnosis of SM with associated MDS/MPN, unclassifiable (MDS/MPN, U) was made fitting the major criterion and one minor criterion for SM and the clinical, morphological and molecular WHO criteria for MDS/MPN, U. Dual-energy X-ray absorptiometry (DEXA) showed lumbar T-Score: −1.5, and total femur and femoral neck (total: +0.2, femoral neck: −1.3) were suggestive for osteopenia that is a typical finding of SM. Since the search for *KIT*D816V mutation was negative, imatinib 400 mg daily was started. After 2 months of therapy, the patient achieved significant improvement in hematological parameters, namely, WBC (7.17 × 10^9^/L) and platelets (177 × 10^9^/L), along with complete resolution of the skin rash. LDH level decreased to 136 IU/L, and tryptase to 3 ng/ml. Due to grade 3 thrombocytopenia imatinib was suspended for two weeks. When platelet count recovered (greater than 100 × 10^9^/L), imatinib was resumed at a lower dose (initially 100 mg daily, and then 200 mg daily). After 3 months of therapy, a new BM biopsy was performed, showing an outstanding response of SM and an improvement in both MDS/MPN and BM fibrosis grade (MF-1) (**Figure 1**). Mast cells decreased up to 1–2%, serum tryptase level on peripheral blood remained within normal range and spleen diameter decreased up to 16 cm. At last follow-up, imatinib was still well tolerated except for mild fluid retention, with no more episodes of hematological toxicity.

## Discussion

Systemic mastocytosis is a heterogeneous hematological neoplasm ranging from indolent to aggressive forms with different prognoses. According to the updated WHO classification, the category of SM-AHN represents a very complex SM variant, depicted by the simultaneous occurrence of hematological clonal non-MC lineage diseases, more frequently myeloid neoplasms in about 85–90% of cases (2, 7).

Although its prognosis depends on both the SM variant and the associated disease, SM-AHN is generally considered to be an advanced variant, multilineage-mutated myeloid neoplasia with a fatal outcome. It is also often difficult to accurately relate the B/C findings to SM or associated disease, and thus the correct classification of the SM variant (8).

Here, we describe the case of a 56-year-old Caucasian woman with *KIT*D816V-unmutated SM associated with a myeloid neoplasm showing overlapping features of both MDS and *BCR-ABL1*-negative MPN. In detail, BM biopsy sample was hypercellular with increased granulopoiesis and loose and dense clusters of megacaryocytes with MPN- and MDS-like characteristics. Additionally, along with splenomegaly, complete blood cells examination showed basophilic leukocytosis with thrombocytosis and mild anemia, allowing us to make a diagnosis of SM associated with MDS/MPN, U versus the alternative option of SSM.

The pathophysiologic basis of the coexistence of SM and other myeloid neoplasms is poorly understood; notably, the driver *KIT* mutation may not be present in the concurrent neoplasm (7). Sotlar et al. investigated the presence of *KIT*D816V mutation in the AHN, showing the coexistence of this mutation in 89% of cases associated with CMML, but only in 20% of patients with SM-MPN (9). Gain-of-function *KIT* mutations lead to a ligand-independent activation of downstream signaling involving PI3K/AKT/mTOR, JAK/STAT, and RAS/RAF/MEK/ERK pathways, promoting proliferation and resistance to apoptosis (10). Non-codon-816 *KIT* mutations have been detected in both patients affected by cutaneous mastocytosis and SM, and they may facilitate the transformation into an aggressive form (3). Overall, *KIT* mutations are detected in over 80% of all SM, nevertheless, additional somatic mutations are reported especially in SM-AHN, including *TET2*, *SRSF2*, *ASXL1*, *RUNX1*, *JAK2*, and *RAS* mutations which may impact on survival, leading to a better prognostication and new targeted treatment in SM (11). Several cases with these somatic mutations have been reported in SM associated with MDS/MPN, showing a very dissimilar prognosis (12). A major challenge is how to integrate clinical characteristics, morphology, and genetic to clarify the diagnosis and prognosis of complex SM subtypes (13). Therefore, treatment in SM is highly individualized (1). Nevertheless, their management could be very difficult, and SM-AHN may transform into more aggressive forms (14). *KIT* mutations are a compass and often guide the therapeutic choice. Since our patient did not show *KIT*D816V mutation, treatment with imatinib was started. Indeed, imatinib has already demonstrated high efficacy *in vitro*, inhibiting both wild-type *KIT* and juxtamembrane mutant c-kit kinase activity, but has no effect on D816V mutant variant (15). In a phase II trial 20 patients regardless their *KIT* mutational status were treated with imatinib, however only one *KIT*D816V-negative patient achieved complete remission, with other six patients showing symptoms improvement, among them two patient were *KIT*D816V-positive (16). Altogether, these data suggest that *KIT*D816V mutation is associated with resistance to imatinib, while, as in our patient, it could represent a suitable approach in *KIT*D816V-unmutated cases, even in those associated with MDS/MPN.

## Data Availability Statement

The original contributions presented in the study are included in the article/supplementary material. Further inquiries can be directed to the corresponding author.

## Ethics Statement

Written informed consent was obtained from the individual(s) for the publication of any potentially identifiable images or data included in this article.

## Author Contributions

All authors listed have made a substantial, direct, and intellectual contribution to the work and approved it for publication.

## Funding

The only funds used were those provided by the authors’ institutions.

## Conflict of Interest

The authors declare that the research was conducted in the absence of any commercial or financial relationships that could be construed as a potential conflict of interest.

## Publisher’s Note

All claims expressed in this article are solely those of the authors and do not necessarily represent those of their affiliated organizations, or those of the publisher, the editors and the reviewers. Any product that may be evaluated in this article, or claim that may be made by its manufacturer, is not guaranteed or endorsed by the publisher.
